# Automated Analysis of a Diverse Synapse Population

**DOI:** 10.1371/journal.pcbi.1002976

**Published:** 2013-03-28

**Authors:** Brad Busse, Stephen Smith

**Affiliations:** 1 Biophysics Program, Stanford University, Stanford, California, United States of America; 2 Program in Physical Biology, Eunice Kennedy Shriver National Institute of Child Health and Human Development, National Institutes of Health, Bethesda, Maryland, United States of America; 3 Department of Molecular and Cellular Physiology, Stanford University, Stanford, California, United States of America; Allen Institute for Brain Science, United States of America

## Abstract

Synapses of the mammalian central nervous system are highly diverse in function and molecular composition. Synapse diversity *per se* may be critical to brain function, since memory and homeostatic mechanisms are thought to be rooted primarily in activity-dependent plastic changes in specific subsets of individual synapses. Unfortunately, the measurement of synapse diversity has been restricted by the limitations of methods capable of measuring synapse properties at the level of individual synapses. Array tomography is a new high-resolution, high-throughput proteomic imaging method that has the potential to advance the measurement of unit-level synapse diversity across large and diverse synapse populations. Here we present an automated feature extraction and classification algorithm designed to quantify synapses from high-dimensional array tomographic data too voluminous for manual analysis. We demonstrate the use of this method to quantify laminar distributions of synapses in mouse somatosensory cortex and validate the classification process by detecting the presence of known but uncommon proteomic profiles. Such classification and quantification will be highly useful in identifying specific subpopulations of synapses exhibiting plasticity in response to perturbations from the environment or the sensory periphery.

## Author Summary

Synaptic connections are fundamental to every aspect of brain function. There is growing recognition that individual synapses are the key sites of the functional plasticity that allows brain circuits to store and retrieve memories and to adapt to changing demands and environments. There is also a growing consensus that many neurological, psychiatric, neurodevelopmental and neurodegenerative disorders may be best understood at the level of specific, proteomically-defined synapse subsets. Here, we introduce and validate computational analysis tools designed to complement array tomography, a new high-resolution proteomic imaging method, to enable the analysis of diverse synapse populations of unprecedentedly large size at the single-synapse level. We expect these new single-synapse classification and analysis tools to substantially advance the search for the specific physical traces, or engrams, of specific memories in the brains synaptic circuits. We also expect these same tools to be useful for identifying the specific subsets of synapses that are impacted by the various synaptically-rooted afflictions of the brain.

## Introduction

Synapses are fundamental to every aspect of brain function. They are recognized today as being highly complex structures and highly diverse in both function and molecular composition. At the structural level, individual synapses of the mammalian central nervous system are thought to comprise hundreds of distinct protein species [1]–[3], and genomic and gene expression data available implies very strongly that there are multiple isoforms of many of these proteins and that their expression is differentially patterned across the brains diverse cell types [4]. It thus seems inescapable that synapses of the brain, even within traditional transmitter-defined synapse categories (e.g., glutamatergic, GABAergic, cholinergic, etc.), must be highly diverse in protein composition [5]. This conclusion is consistent with the available functional data, where physiological studies report wide differences in synaptic transmission as different brain regions and pathways are examined (again, even when results are compared only within traditional neurotransmitter categories). Moreover, the well-known functional plasticity of both synapse structure and synapse function in response to electrical activity implies directly that even an otherwise homogeneous synapse population must become heterogeneous or diverse after individual synapses experience differential activity. In this light, it seems likely that synapse diversity per se may be critical to the proper function of neural circuitry. For instance, there is now widely believed that the plasticity (and therefore resulting diversity) of individual synapses is fundamental to memory storage and retrieval and to many other aspects of neural circuit adaptation to environmental change [6], [7].

Unfortunately, the measurement of synapse diversity has been restricted by the limitations of available methods capable of resolving individual synapses. Array tomography (AT) is a new high-resolution, high-throughput proteomic imaging method that has the potential to very substantially advance the measurement of unit-level synapse diversity across large and diverse synapse populations. AT uses multiple cycles of immunohistochemical labeling on thin sections of resin-embedded tissue to image the proteomic composition of synapse-sized structures in a depth-invariant manner. We have applied AT to freshly-fixed mouse cerebral cortex, where our volumes have typical sizes of thousands to millions of 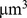 of tissue, contain millions of individually-resolved synapses, and label over a dozen multiplexed proteomic markers.

With proper analysis, the informational density of array tomographic volumes has numerous potential applications. Synapse-level resolution of large volumes of tissue is an ideal tool for addressing interesting hypotheses concerning principles like synaptic scaling [6], structural arrangement [8], and novel synapse types [9], [10]. Combined with connectomic data [11], [12], genetic models [13], [14] or dye filling techniques [15], [16], array tomography can also address questions regarding proteomic distributions in specific subsets of cells. We are interested in investigations of this nature and others in the mouse cerebral cortex, where the anatomical distribution of synapses, aside from cortical layer cytoarchitectonics, is currently largely unexplored.

### Developing a Method of Synapse Quantification

**Figure 1:**
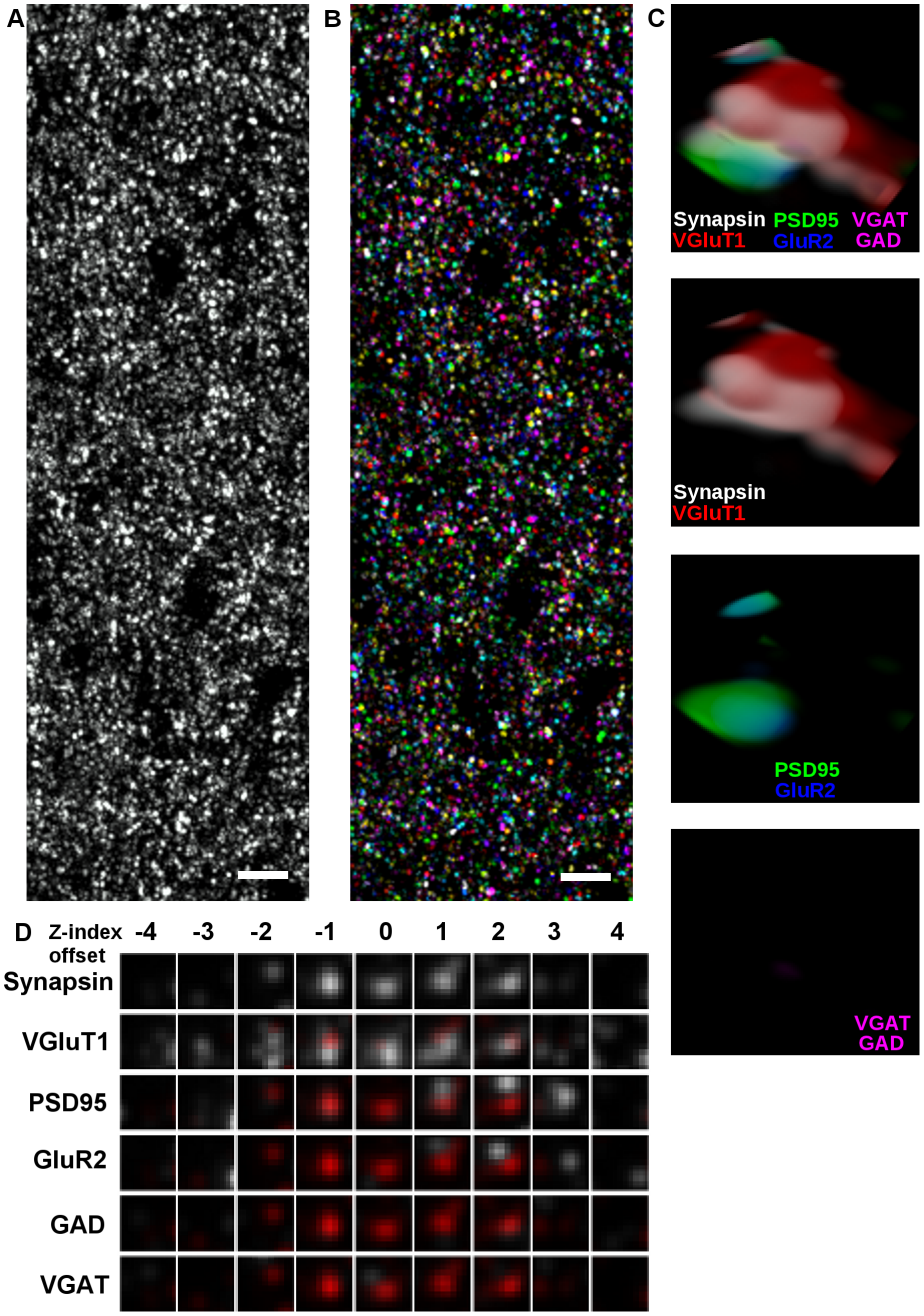
The synaptogram as a tool for high-dimensional proteomic visualization. (A) A maximum projected volume of Synapsin I labeling. 41 slices, 70 nm per slice, total thickness of 2.87 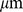. (B) Randomly-colored segmentation of individual synapsin puncta. (C) Rendering of a single punctum from the volume showing synapsin (white), imaged together with VGluT1 (red), PSD95 (green), GluR2 (blue), GAD (magenta) and VGAT (magenta). From top to bottom: all proteomic markers, glutamatergic presynaptic labels, glutamatergic postsynaptic labels, GABAergic labels. This appears to be a glutamatergic synapse. (D) The synaptogram derived from the same synapse. Synapsin, top row, is repeated in red for the rest to provide spatial context. Not shown, sixteen other colors and two redundant labels (synapsin and VGluT1). Scale bar: 5 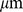, size of synaptogram/render volume, 1100 nm × 1100 nm × 630 nm.

Utilizing array tomography to its fullest extent requires the development of new synapse detection and classification capabilities. Simple analysis, using repeated human observation of a fraction of the channels available in the full volume, may be acceptable for analyzing fragmentary subsets of a few hundred synapses but cannot scale beyond that. We have developed tools and methods to assist in handling the high proteomic dimensionality of array tomographic volumes (Figure 1), principally the synaptogram [17]; a means of visualizing small pieces of highly multiplexed data by splaying out the 3-D volume surrounding a region of interest (ostensibly a single synapse) into a larger 2-D image.

An example of a synaptogram in action can be seen in Figure 1-C,D, both of which visualize the same synaptic volume. 1-C attempts to render the volume in three dimensions, assigning a different color to each channel, and running out of easily separable colors in the process, even for this one example. It also falls prone to the usual pitfalls of obscuration and optical confusion common to snapshots of rendered scenes, such that splitting the image into multiple ones displaying subsets of colors helps visualization considerably. Contrast this with a synaptogram of the same synapse in Figure 1-D. Each row of thumbnails displays a different channel (plus synapsin, included to serve as a reference channel), each column shows a different z-section; left is below, right is above. Unlike the render, position and depth relationships are presented clearly, and the synaptogram can be extended to include an arbitrarily large number of simultaneous imaging channels by appending new rows vertically.

With only a bit of exposure to synaptograms, human experts can use them to tell at a glance exactly what they're seeing. This eases the difficulty of per-synapse manual classification such that the effort of classifying a set of few hundred synapses is no longer excessive, but no matter how convenient they are to analyze individually, the sheer number of synapses makes manual analysis of the entire data set effectively impractical.

Given that just a few hundred analyzed examples can be obtained with a reasonable expenditure of effort, there are two approaches to consider. The first is to use those examples as a representative sample, in a manner similar to stereology. That may work well for some questions, but not others. Rare or novel synapse types and cortical laminar distributions would be especially difficult to study. An alternative, which this paper will present, is to take that sample of accurately classified synapses and extrapolate its decision-making information to the much larger population of unclassified individuals.

## Results

### Identifying Putative Synaptic Loci

The first necessary step in our classification process is to locate the sites which may contain synapses. Despite their appreciable proteomic diversity [18], cortical synapses are small: from the ostensible midpoint of the synapse, all relevant synaptic protein labeling can fit within a 500 nanometer radius for mouse cortex [19]. Given a reliable method of locating synapses, all information needed to verify and type those synapses can be measured from the local volume surrounding them, greatly reducing the spatial analysis needed per synapse. To avoid confusion with actual synapses, we refer to these sorts of putative synapse locations as “synaptic loci.” They are specific places which might be synaptic.

In order to find putative synapses to help limit the necessary search space, we are using an antibody targeting Synapsin I. Synapsin is a scaffolding protein reportedly found in all cortical synapses [20], and labeled antibodies targeting synapsin have previously been used on their own to estimate synapse counts [21]. A Millipore Rabbit anti-Synapsin I antibody (Millipore AB1543P) demonstrates robust and reliable labeling, and is likely to be colocalized with all relevant synaptic markers [17]. For these reasons the core of our analysis uses Synapsin I labeling to derive a list of locations likely to contain synapses from which to begin small volumetric searches for confirmation. Our approach is to use the brightest point of each Synapsin I punctum as the site of a possible synapse to designate a local volume for further analysis, without attempting to explicitly determine the punctum boundaries.

We prefer our local maxima-based approach over thresholding-based segmentation because the latter has a number of issues arising from AT's largely anisotropic resolution (∼200nm × ∼200nm × 70nm). This anisotropy, combined with (often unknown) epitope density and labeling variance means that any segmented punctum boundary is at best an estimate. An approach using local maxima, paired with a voxel-based rotation-invariant feature set, is not affected by the exact boundaries of the puncta of interest, but by the puncta themselves.

While our approach to synapse discovery sidesteps segmentation, it does so at the cost of introducing potential false positives: background local maxima which segmentation would have discarded, but whose peak brightness rises over our low threshold for consideration. However, it is possible to filter those out in later classification. Conversely, this method is ideal for teasing apart “clumps” of synaptic labeling, where multiple synapses exist in close proximity but can be resolved by the Rayleigh criterion and thus having separate local maxima.

### Manual Classification

#### Using human experts

It has been demonstrated that the fluorescently labeled markers used in AT corroborate well with their identification from EM ultrastructure, despite the deleterious effects of glutaraldehyde fixation on tissue antigenicity [17]. It then follows that a process by which synapses can be reliably identified via their component markers imaged in light microscopy alone will inherit the corroboration and can be said to be faithful representations of synapse populations.

Humans can visually identify the synaptic category of a given locus via the use of synaptograms (Figure 1), using the spatial juxtaposition of a number of relevant synaptic molecules for classification [17]. Glutamatergic synapses, for example, will by definition have at least one vesicular glutamate transport protein and at least one post synaptic density scaffolding protein present. Similarly, GABAergic synapses can be identified by the presence of glutamic acid decarboxylase (GAD) and a vesicular GABA transport protein.

This process of human synapse identification is the best and most reliable method of synapse identification available to us. It relies on the perception and expertise of the human viewer to apply the visual segmentation which defines the “presence” of necessary labels. This task incorporates a great deal of *a priori* knowledge concerning the stearic and functional relationships between the different molecular labels, the variance in labeling of each particular antibody, and the particular conditions under which that tissue had been fixed, embedded, labeled, imaged, relabeled, etc.

Although manual classification of fluorescence data is orders of magnitude faster than EM stereology, it is still orders of magnitude slower than needed to keep up with the synaptic output rate of AT volumes. For that, we decided to use human-generated classifications as training data, then liberally applied a number of clustering and supervised learning methods to quantitatively mimic the human decision making process. Assuming the machine learning algorithm thus trained could perform on par with a human expert it can then be used in place of the human, though to avoid “garbage-in, garbage-out” scenarios, this does not obviate the need for error estimation when faced with novel problems.

### Machine Learning

Machine learning methods come in two broad categories. Supervised learning algorithms, trained using a sufficient number of human rated synapses, are capable of producing numerical descriptions of human judgment as it is applied to synapse classification, as well as extrapolating that judgment to the hundreds of thousands of synapses which comprise an average data set. Unsupervised clustering, on the other hand, when applied to raw synaptic loci or already classified synapses is a great approach to the discovery of marginal classes or subtle subtypes.

#### Feature extraction

The first step in constructing a computational framework for either form of synapse classification is to find a set of explicit measurements which span the feature space that human raters implicitly search. We are using a small set of ad hoc, channel-independent, rotationally invariant features to measure the spatial distribution of each channel's fluorescence about the synaptic locus. These features are calculated per voxel, without relying on segmentation, combinatorial information or *a priori* geometrical information, in keeping with the rationale behind finding the loci in a similarly parameter-independent manner. The equations used to calculate the four features are given below.

For every voxel *i* in the local 11×11×11 voxel window *V* with brightness *b* and pixelwise distance from the synaptic locus *d*:
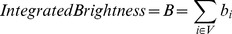
(1)

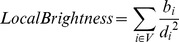
(2)

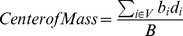
(3)

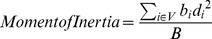
(4)Of these features, the Integrated Brightness is the simplest to describe, as it is the sum of all the pixel values within 5 pixels. Local Brightness is also the sum of all values within 5 pixels, but the contribution of each pixel is reduced by the square of its distance from the locus. It can be used as a metric for estimating the volume of the punctum without segmentation because nearby pixels (more likely to be part of the punctum) contribute much more heavily than distant ones (more likely to be noise or neighbors). To test this assumption, we compared scores produced by this feature to that of a simple connected component analysis measuring size directly, and found a high degree of correlation (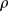 = 0.829). The remaining features, Center of Mass and Moment of Inertia, treat the puncta brightness as if it is a mass distribution in a synaptogram-sized object, and respectively compute the distance to the center of that object and its angular inertia for a rotation about the locus. The combination of all four features effectively describe the fluorescence distribution in a synaptogram.

The result of this feature extraction, when performed on a multidimensional image of *c* channels, is a 4*c*-long numerical vector of proteomic measurements describing the putative synapse. This analysis is repeated for each of *p* synaptic loci in the data set, giving us a *p *× 4*c* matrix of measurements to be further analyzed. To enhance consistency between data sets, which may well have different imaging conditions, we normalize each of the extracted features by dividing by the population's mean score.

### Clustering

Although visual analysis is the traditional and preferred method of examining biological data, long strings of numbers such as our feature vectors are difficult for humans to visualize. In response, high-dimensional numerical measurements have often been approached using some form of dimensionality reduction as a first step in numerical analysis. Simply put, reducing a long string of numbers to a short string of numbers makes them easier graphically display and understand. Principal Component Analysis (PCA) is a venerable method of dimensionality reduction which has seen use in similar applications [22], [23], and has proven useful in ours as well.

**Figure 2:**
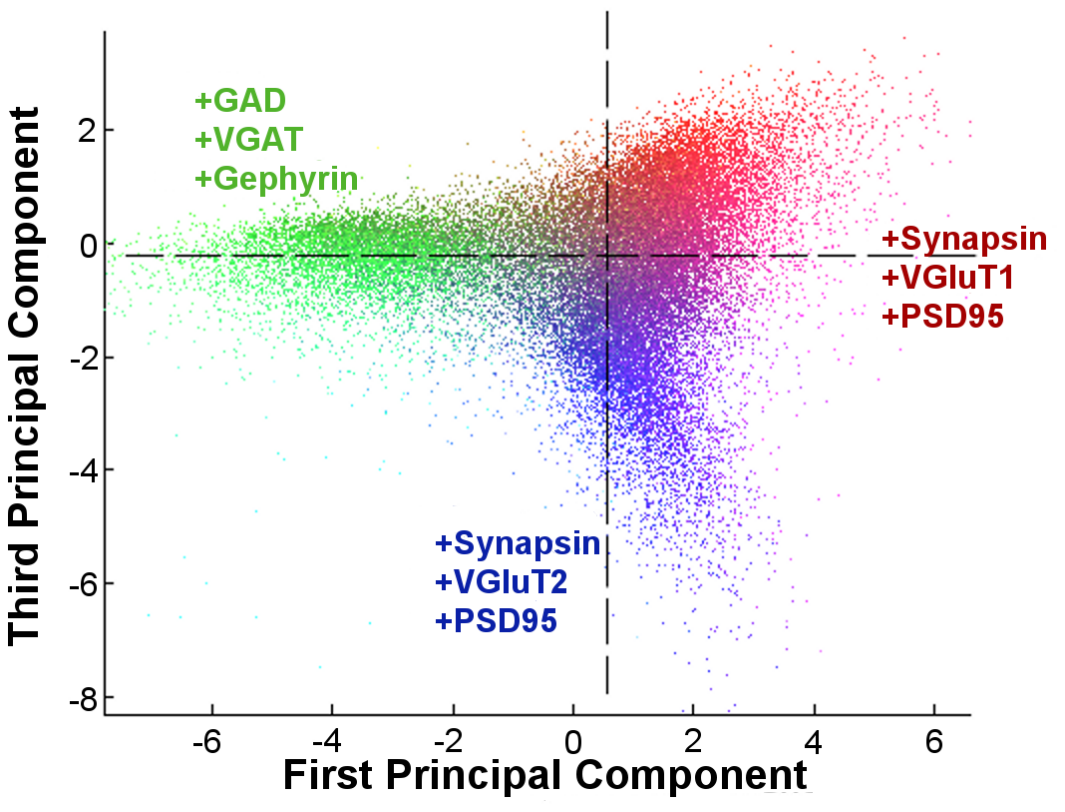
Clustering of synapsin I imaged with array tomography. When the first and third principal components of the local brightness feature eq 1 are plotted against each other, they form clusters identifiable as known synaptic subtypes, and explain 50.4% of the variance in the data.

Our PCA result, illustrated in Figure 2, identifies some synaptic populations but does not separate them sufficiently for classification. The loci tend to aggregate in clusters which correspond to a few of the broader synaptic categorizations, namely GABAergic and two common subtypes of glutamatergic synapses. We identified the clusters using multivariate regression, that is, taking a few of the more distant examples and inferring the contribution of channels which brought them from the mean. The PCA demonstrates the existence of separate populations corresponding to each class, but in the reduced dimensionality of PCA, simple thresholds are insufficient for proper class discrimination. The two components plotted explain 50.4% of the variance between them. An additional 20.9% of the variance is present in the second principal component (not shown), which appeared to represent synapse size.

Ideally, the dimensionality reduction accomplished by the above methods would have proven amenable to simple thresholding. If that where the case, multivariate regression might have led to identification and, combined with a measure of the statistical significance of cluster separation, classification of unknown synapses based solely on where they fell in the unsupervised plot. Since our clusters were not so cleanly separable, we resorted to a more subtle stratagem involving supervised learning.

### Classification

The “supervision” of supervised learning refers to the supervised training set, a random or semi-random collection of human-rated examples from which the machine learning algorithm (MLA) infers the rules for classification to extrapolate onto novel synapses. To generate each item of the set, we presented a synaptogram to a human trainer, who rated the synaptogram in one or more binary categories representing the presence or absence of channels relevant to synapse classes of interest. We could then associate those categorizations with the already-derived feature vectors of those examples, compiling them into a library of “correct” classifications for training.

#### MLA selection

Another necessary choice in supervised learning is that of the MLA used as a classifier. In an early training experiment, we created a training set of 200 examples classified into glutamatergic/non-glutamatergic and GABAergic/non-GABAergic categories. We fed these results into an assortment of MLAs with minimal parameter optimization. The error rates of the various MLAs are presented in Table 1. Although many of these algorithms performed well, combined with the posterior metrics detailed below, the random forest ensemble [24] slightly edged out the competition and earned its place as our classifier of choice. One observation worth mentioning: all classifiers performed better when detecting GABAergic synapses, as compared to glutamatergic. At the time this comparison was conducted, we had presumed the systematic error to stem from the reduced background staining of our GABAergic channels. However, after conducting our channel-based classification comparison (below), we currently suspect it has more to do with the comparatively higher classification error of PSD95.

**Table 1:** Machine Learning Algorithm comparison.

	LDA	QDA	NB	NBkd	RFE	kNN	SVM
Glut	0.128	0.114	0.110	0.104	0.084	0.176	0.222
GABA	0.044	0.036	0.062	0.052	0.036	0.070	0.178

Comparison of various supervised machine learning algorithms. A small training set was used to compare the error rates of multiple MLAs when classifying glutamatergic and GABAergic synapses in an early data set. From left to right: Linear Discriminant Analysis (LDA); Quadratic Discriminant Analysis (QDA); Naive Bayesian filter, gaussian distribution assumption (NB); Naive Bayesian filter, normalized kernel distribution assumption (NBkd); Random Forest Ensemble (RFE); k-means clustering (kNN); Support Vector Machine (SVN). k-means clustering, an unsupervised clustering method, was included for comparison's sake.

#### Global feature importance

An additional point in favor of the random forest ensemble was the useful posterior metrics which can be simply derived as a byproduct of its algorithmic structure. Posterior metrics are methods of analyzing the process of classification after classification. Their primary purpose is to relate information about why a given locus was classified one way or another, and meta-information such as the relationship between classes and the features which proved more important than others during classification.

Each decision tree in a random forest is a series of optimal feature threshold branches with decisions for leaves. By keeping track of which feature was used for each branch point, along with the confidence that branch point engenders, we could gauge the importance of the various features relative to each other. Overall, our local brightness feature proved most useful, with the rest decreasing in performance. Normalized to the local brightness importance, feature values were 0.76, 1.00, 0.59 and 0.55 for the integrated brightness, local brightness, center of mass and moment of inertia features, respectively. Though the local brightness feature may have outshone the others, all proved useful in classification.

#### Channel-based classification

For our preliminary analyses, including the MLA choice analysis and the human agreement test, we classified synapses based on specific synapse classes: a synaptic locus was marked positive if and only if its synaptogram included all the requisite markers for a synapse of the class in question. Iterated over an entire data set, a human expert looking for this synaptic class (or an algorithm trained to do the same) should be able to identify all such synapses. While this approach adroitly combined the tasks of synapse identification and classification for this particular type of synapse, it could not serve as a general method for synapse identification. Novel synapse types with unexpected channel combinations were likely to be discarded as noise, if missing a critical channel, or else lumped in with existing synapse types.

In order to facilitate the discovery of novel synapse populations, we decided to split the classification of loci into multiple parallel classification steps. Rather than train one MLA per synapse class to detect the combination of all relevant channels, we trained one MLA per channel to detect the “presence” of that channel at each locus, and allowed the combination of their predictions to identify synapses of that class. For example, rather than training a glutamatergic synapse classifier to detect glutamatergic synapses, we used individual classifiers for the relevant channels (VGluT1, VGluT2, PSD95), and then combined their outputs in the same logical way ((VGluT1 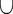 VGluT2) 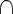 PSD95) to identify glutamatergic synapses.

Approaching the problem of synapse classification in this manner imparts several benefits to our process. Principally, it facilitates the identification of novel synapse types by allowing us to quickly recombine classified channels. For example, if for some reason we suspected the existence of VGAT-positive glutamatergic synapses, it would be simple to add a 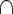 VGAT term to the above logical condition for glutamatergic synapses, and see if the resulting population occurs significantly above chance.

An additional but perhaps more fundamental benefit of our channel-based approach is its greater resemblance to the method by which AT labeling can be validated with EM [17]. If desired, the output of a channel-classifier can be compared directly to the EM with a single immunolabel, as opposed to the three or so needed to verify the output of a full synapse classifier.

#### Active learning and rare classes

In most supervised learning models, training set examples are sampled entirely at random in order for the training set to have the same statistical properties of the full data set. This can be inefficient for us in the of case of uncommon channels. The less common a given channel is, the more negative results a human has to sort through before reaching a usable number of positive results. For example, VGluT3 positive loci can be identified in much the same manner as VGluT1 or VGluT2 loci, but due to their paucity in the cortex (we see roughly 1.2 VGluT3+ loci per one thousand negative loci), human raters would have to classify excessive numbers of negative loci for each positive locus in the training set.

In order to address this possibility, our classification process is a two-phased nonrandom selection of training examples. It is described in detail in the methods section but, briefly, functions by actively using the classifier it is training to select examples that help ensure a diverse training set, and presents each example's predicted class to the user. The net effect of the training modification is to focus the human role more on verification and correction than strict instruction. Aside from accomplishing the goal of efficiently training classifiers for rare classes, we find that the active version seems to be much less of a strain on human patience than *de novo* training, even that aided by synaptograms. It also reduces the necessary training set size to roughly twice the number of requisite positive synapses in the training set, despite the rarity of the class in question.

Once the human raters are satisfied with their training sets, we pass the entire data volume through the classifiers for identification, and collate the results into a combinatorial set of vectors.

### Post-Classification Analysis

After classification, the predicted presence of each channel for a given locus can be derived from the percentage of decision trees in the random forest ensemble which attest to its presence. This effectively serves as a confidence metric for the entire ensemble, and is generally referred to as the “posterior probability.” An instance with a posterior probability of 1.0 is unequivocally positive for the class in question, one of 0.0 is undeniably negative. In this manner, we reduce the 4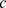-long numeric feature vector to a 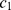-long numeric posterior vector, representing the presence or absence of all 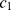 relevant channels. We can then use these vectors in a combinatorial fashion to recreate synaptic classes. Glutamatergic VGluT1-expressing synapses, for example, should at a minimum be positive (posterior probability 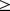
0.5) for VGluT1 and PSD95.

#### Per-channel feature importance

**Figure 3:**
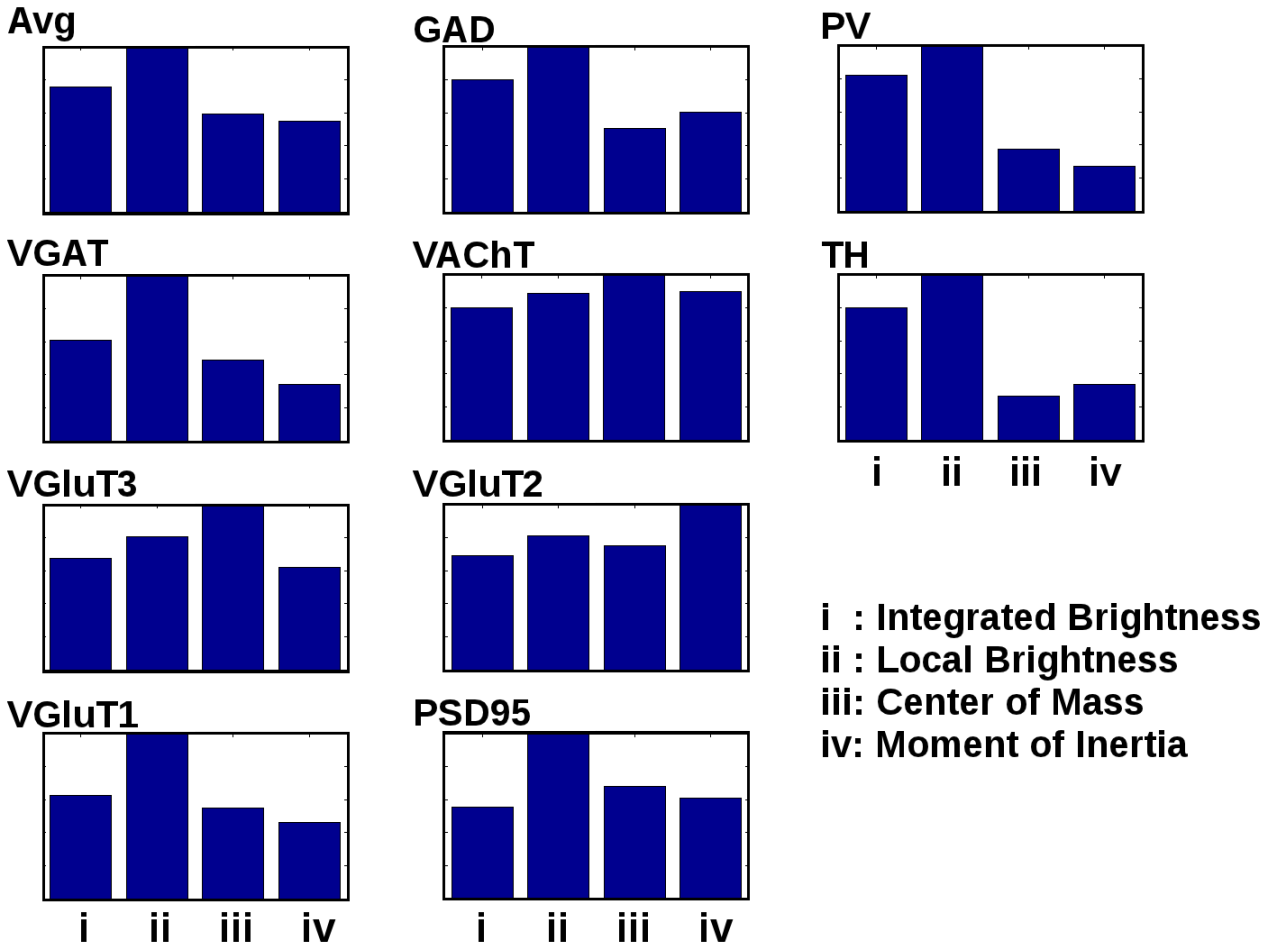
Relative feature importance for different molecular labels. When all classes were averaged (top left), our local brightness feature (ii) saw the most use, followed by integrated brightness (i), center of mass (iii) and moment of inertia (iv). GAD, VGAT, PV, VGluT3, VGluT2, VGluT1, PSD95, VAChT, and TH each make slightly different use of the feature set. VGluT3, VGluT2, and VAChT are notable in that they rely most heavily on features other than local brightness.

Since our labeled channels occupy a number of spatial niches in the canonical synapse, we were interested in determining which features contributed most to which channel classifier, in case that reflected the differential distribution. The results are shown in Figure 3. The channels which differ from the norm (Figure 3-A) in selecting the center of mass or moment of inertia features as their most important included VGluT2, VGluT3 and VAChT. These channels are all presynaptic, which eliminates spatial differentiation as a cause, but interestingly they are all uncommon to rare. TH, also rare, did not display this behavior, and also differs from the rest in that “neighboring” puncta were deemed acceptable for positive classification. This may suggest that for rare classes where neighbor discrimination is important, determining whether a discovered punctum is part of the synapse in question or a close neighbor plays a bigger role in the accuracy rate than discovering the punctum in the first place.

#### OOB error as cross-validation step

The training process of the random forest classification itself provides a reliable approximation of its error rate. During training, each tree in a random forest excludes a random fraction of examples from its construction, which can later be used in the manner of cross-validation testing to gauge the accuracy of that tree. More precisely, each training example can function as withheld data for a sub-random forest ensemble composed of the fraction of decision trees to have excluded it during training, and, taken in aggregate, are an estimate of the performance of the full forest. This is called the “out-of-bag error” [24]. OOB performance for the classes we are interested in can be found in Table 2. The OOB error can be interpreted as a self-estimation of the classifier's true error rate. Of note is PSD95, with an error rate as high as the rarer classes, probably due to its postsynaptic location whereas all other markers are presynaptic.

**Table 2:** MLA training comparison.

Channel	OOB Error
GAD	0.0400
VGAT	0.0767
PV	0.0814
VGluT3	0.1146
VGluT2	0.0716
VGluT1	0.0690
PSD95	0.1215
VAChT	0.1394
TH	0.0333

Out-of-bag (OOB) error rate estimates for various classified markers. Order of markers is the same as in Figure 6. Each classifier had a minimum training set size of one hundred examples.

#### Comparison to human rating

**Figure 4:**
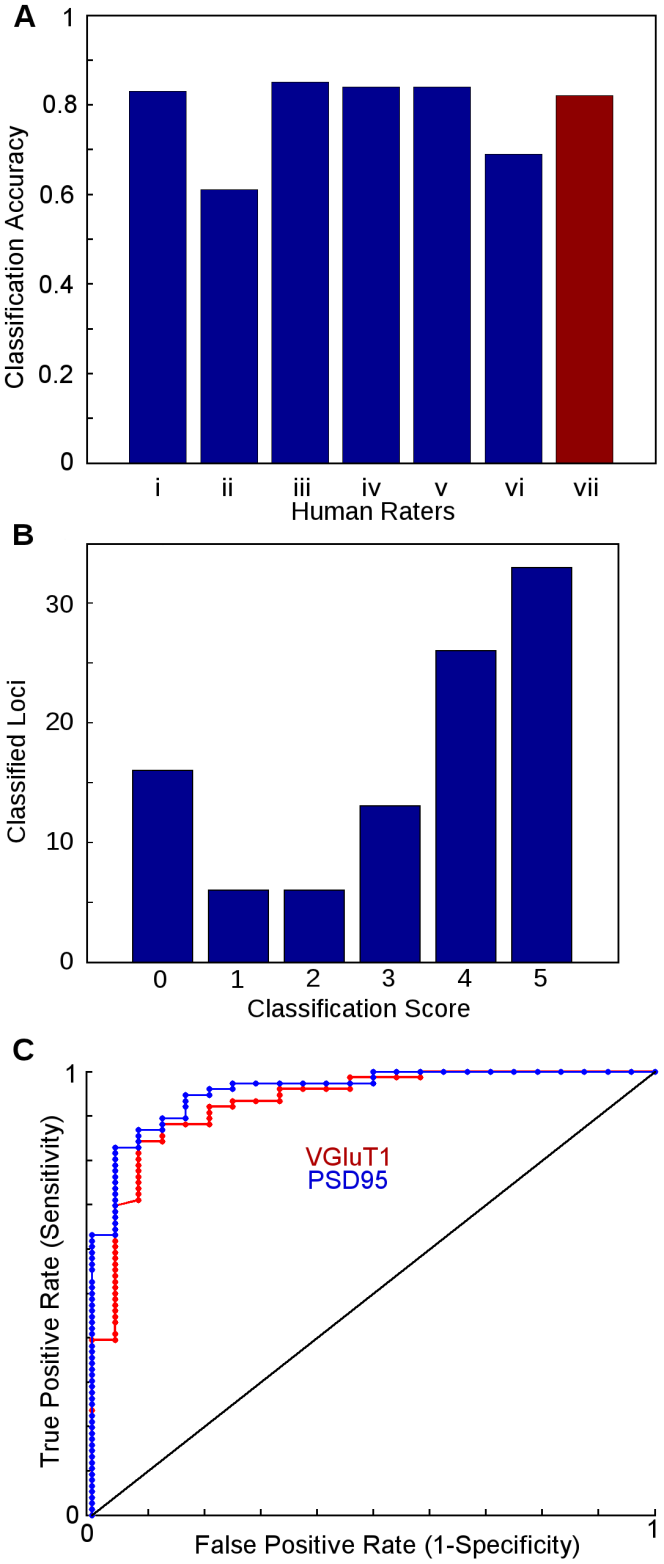
Comparison of human rating to machine learning. (A) Accuracy rates. **i-vi** - When compared against the average decisions of their peers in a VGluT1 synapse discrimination task, humans performed at different accuracy levels based on their stringency of classification. **vii** - The random forest ensemble, (VGluT1 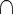 PSD95), trained by human rater i, performed comparably to the humans. (B) Rater agreement histogram. 100 individually-classified synaptic loci are scored according to the number of “yes” votes received among the five humans composing the gold standard consensus. Situations with unanimous agreement (0,5) make up half of the set (49 loci), with an additional 37 examples having only one dissenting opinion (1,4). (C) Receiver operating characteristics (ROC) curve, for VGluT1 and PSD95 classifications on human-rated data. The ROC curve describes the tradeoff between reducing false positives (left side of the curve) and maximizing true positives (right side of the curve). The displayed diagonal line represents chance, with better classifiers occupying large areas between the diagonal and their own curves.

To quantitatively examine this system's performance when applied to real synapse classification, we ran our human accuracy test set through the VGluT1 and PSD95 classifiers, then compared the combined output (VGluT1 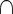 PSD95) loci with that given by humans. Although these two channels had the worst OOB performance, the intersection of the two was about as accurate as the best human raters. We performed a receiver operating characteristics analysis to describe the classifier performance in a more detailed fashion; it is shown in Figure 4B. The fact that the worst OOB error is still equal to the agreement of human raters implies the output of the classifiers should be usable with the same degree of confidence as that of human raters.

### Classification Application

#### Synapse class definition

The use of a channel-based classification process allows us somewhat greater flexibility in the definition of synaptic classes. Our lab has years of experience in recognizing VGluT1-glutamatergic, VGluT2-glutamatergic and GABAergic synapses [17], which compose the majority of synapses in the cortex, and all are defined by the presence of at least two specific markers, in addition to Synapsin I. For this paper we have also included a number of labels targeting synaptic populations for which we haven't found a robust label for a ubiquitous second protein. This includes VGluT3-positive synapses, cholinergic (vesicular acetylcholine transporter [VAChT]) and dopaminergic/noradrenergic (tyrosine hydroxylase [TH] positive) synapses. It is our intention to find such corroborating labels before these channels are used in a full experiment. Additionally, dopaminergic synapses have been reported not to express much of the Synapsin I/II isoforms, if they express them at all [25]. Since we are using a Synapsin I marker to discover putative synapse loci, those which are positive for TH may actually be identifying simple synaptic complexes - dopaminergic synapses adjoining those of another class.

#### Cortical depth analysis

**Figure 5:**
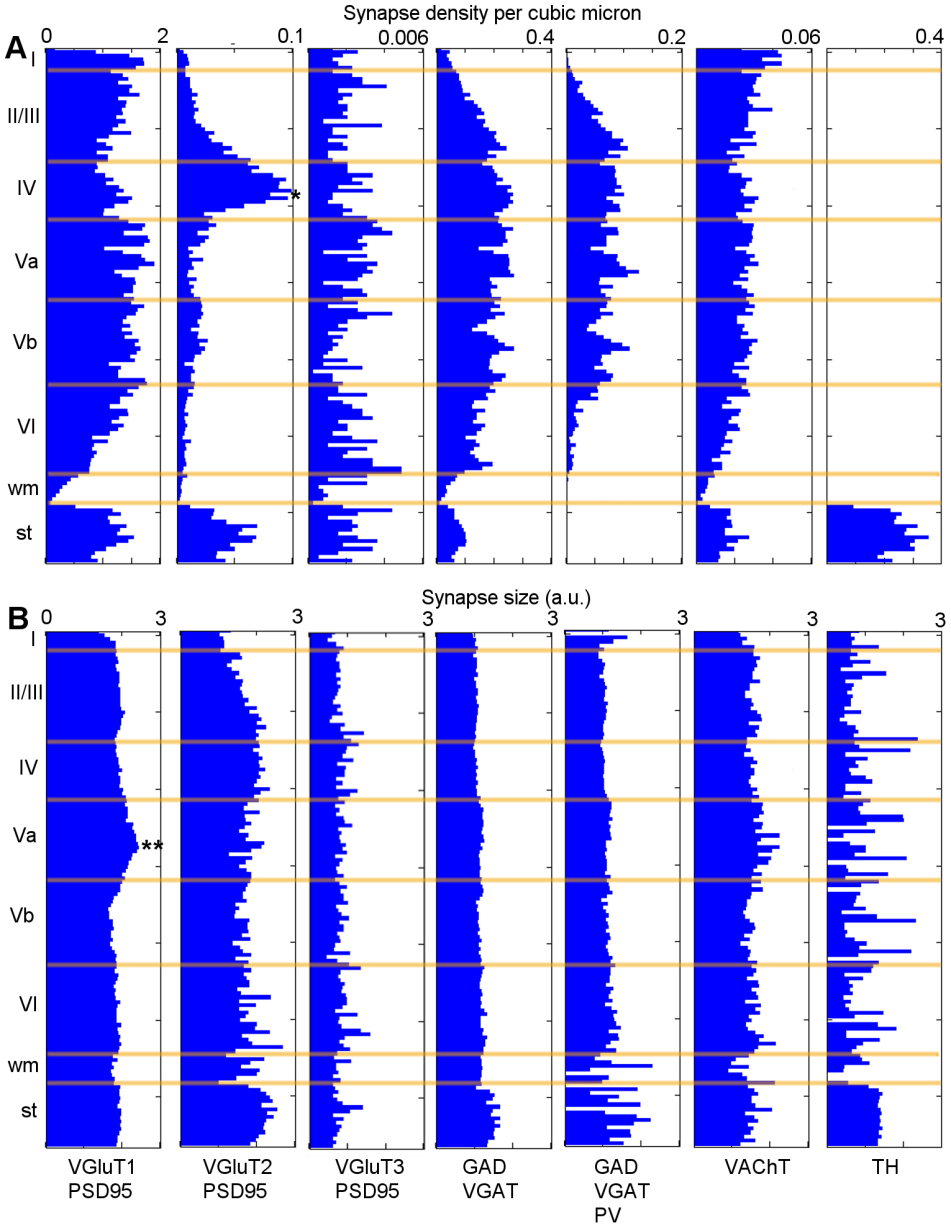
Density and size of synapse classes as a function of depth through the cortex. (A) Synapse density through the cortex. * - VGluT2 synapse density peaks in layer IV. PV-positive GABAergic synapse density is slightly decreased in layer I, and significantly lacking in layer VI. (B) Synapse size estimated using the synapsin local brightness measurement. ** - VGluT1 size peaks in layer Va (p
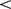
0.05).

One straightforward application of synapse-classified array tomography can be had via cytoarchitectonics, as seen in Figure 5. We first segregated the data into a number of synaptic classes, then subdivided those into 10 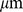 bins stretching from the pial surface of the cortex to the striatum. We calculated the density of each bin's population, and averaged the Synapsin I local brightness feature to estimate the mean synapse size. Overall, the synaptic densities were nearly twice as high as expected in the literature [26], likely due to tissue shrinkage during dehydration and LR White embedding [27]. To be certain, we used erythrocyte diameter measurements to estimate the tissue shrinkage of this block at 56% (Figure S1).

Although larger samples and higher sample sizes will be necessary for statistical certainty of novel phenomena, there are a few interesting known observations which help to validate the method used here. First, there is an increase in VGluT2-positive synapse density in layer IV, which we expected given the laminar characterization of VGluT2-expressing synapses [17]. Second, we notice a decrease in the density of parvalbumin-positive GABAergic synapses in layers I and VI, similar to [28]. Finally, we find that VGluT1-positive synapses in layer 5a, though not more dense than elsewhere in the cortex, are somewhat larger.

In order to gauge the repeatability of this analysis method we confirmed the most prominent of these effects, the spike in VGluT2-positive synapse density in layer IV, using data taken from a mouse whisker barrel (courtesy of Nicholas Weiler, unpublished). This is presented in Figure S2.

#### Pairwise proteomic analysis

Another promising possibility is the use of data sets classified in our per-channel fashion to search for unexpected proteomic combinations which may correspond to novel synaptic subsets, particularly of rare classes. In any volume, some background noise is to be expected: given the spatial distribution of synapses, it is inevitable that some synapses will have asynaptic puncta, or those belonging to nearby synapses, expressed in the region of analysis. Assuming that two classified markers have independent distributions, the expected number of loci in a volume which will be classified positive for both is the product of their probabilities, 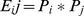. We can compare this with 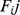, the number of colocalized loci actually found in the data set, and use a two-tailed binomial test to check for significance and reject stochastic noise as an explanation.

For example, VGluT3 has previously been intimated to be present in a very small subset of cortical GABAergic synapses [29]. Since we have labeled both GABAergic synapses and VGluT3 puncta in the course of classifying their respective categories, we can simply retain those GABAergic synapses which classed VGluT3-positive. A two-tailed binomial test can tell if the overlap we observe (82 synapses) is significantly different from that we would expect by multiplying the two class probabilities together (43 synapses). Those are small numbers in a data set of nearly a million classified synapses, but the difference between them is significant (p
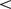
0.001).

**Figure 6:**
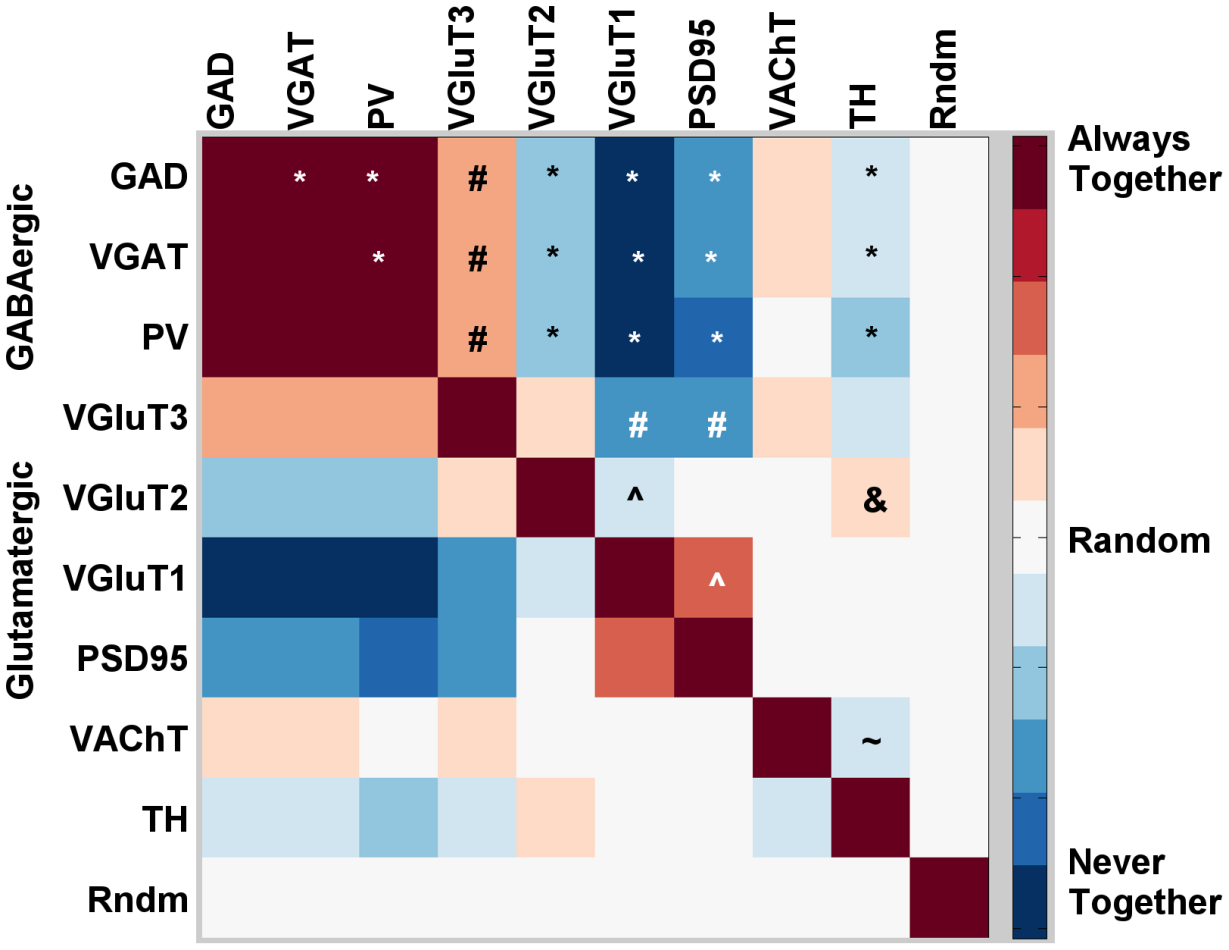
Positive and negative pairwise channel copresence. Symbols denote interesting comparisons with statistical significance of 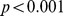. Red squares represent label pairs which are copresent more than expected, blue squares less than expected by chance. * - GABAergic markers are copresent with each other, but avoid glutamatergic and TH markers. ** ?** - VGluT1/2 are copresent with PSD95, but not with each other. **#** - VGluT3 is present with all three GABAergic markers, but avoids VGluT1 and PSD95. & - VGluT2 shows some presence with TH. **∼** - TH tends to avoid VAChT.

Using the nine classified channels in our present analysis, we ran binomial tests to calculate the normalized pairwise relationship between each of them. Our results are presented in Figure 6. The significant results match our expected relationships for the most part - GAD, VGAT, parvalbumin all colocalize, as do VGluT1/PSD95 and VGluT2/PSD95, and all three categories are mutually exclusionary. There are a few points of interest - as mentioned, VGluT3 colocalizes with all GABAergic channels and excludes itself from PSD95, corroborating the literature's suggestion of VGluT3 as a supporting neurotransmitter and not a primary glutamatergic synapse class on its own [30]. Additionally, TH generally avoids both VGluT1 and VAChT, but shows positive copresence with VGluT2 (though this relationship disappears in the striatum).

## Discussion

### Synapse Class Discovery

When we began the class discovery process as shown in Figure 6, we expected relationships based on our preconceived notions of a few synapse classes: that GAD, VGAT and parvalbumin should all be copresent to some extent [28], and that VGluT1 and VGluT2 should each colocalize with PSD95, but not with each other [31]. Since the algorithm was partially developed with these relationships in mind, it is unsurprising that we found them.

The other channels we examined, VGluT3, VAChT and TH, had fewer performance expectations, largely due to their paucity in the data set. Of the three, VGluT3 displayed the most interestingly unexpected behavior, avoiding the common glutamatergic markers and colocalizing instead with all GABAergic synaptic markers. This is most likely a rare but suspected role of VGluT3 in the cortex [30], co-expressed with GAD in a minor population of excitatory interneurons. If so, a followup experiment with larger volumes may be able to more effectively study this sparse population, but its detection given even a small number of examples lends us a degree of confidence that our analysis returns usable results sufficient to detect novel synaptic phenomena.

Another interesting result with even fewer candidate synapses is a cortex-only localization of VGluT2 and TH. Dopaminergic (TH) neurons have been reported to express VGluT2 in rat cultures [32], midbrain and hypothalamus [33]. It is possible that these are afferent projections from subthalamic nuclei, in which case their localization within the cortex and further proteomic differentiation would be interesting to examine in more detail. However, with current volume sizes we only find a dozen of these appositions, so at this time it would be problematic to assert certain confirmation of their existence, much less their distribution.

### Human Consensus Formation

The variance of human raters raises a few interesting questions to look into in the future. Two of the six raters (#2 and #6) self-reported using a stricter standard of classification than the rest: when an example was at all doubtful, they classified it as being negative. Effectively, these raters elected to position themselves on the left side of the ROC curve, trading an increase in false negatives for reduced false positives. Depending on the application, stricter classification may be preferable. We tested an example of this sort of premeditated error ourselves by training a number of MLAs with various classification criteria, and comparing their output. These results are presented in Table 3. As one might expect, we found that a bit of bias in the training process could go a long way to reducing either Type I or II errors, at the cost of increasing the other, and that this effect is exaggerated when processing examples human raters find difficult.

**Table 3:** Estimated error rates for different training models.

All Examples		
Strategy	False Positive	False Negative
Normal	0.006	0.003
Gullible	0.142	<0.001
Cynical	<0.001	0.212

Easy Examples		
Strategy	False Positive	False Negative
Normal	0.007	0.004
Gullible	0.104	<0.001
Cynical	<0.001	0.146

Hard Examples		
Strategy	False Positive	False Negative
Normal	<0.001	0.001
Gullible	0.250	<0.001
Cynical	<0.001	0.398

Type I and II error breakdown for various training regimes. (Top) Classifiers trained with varying approaches to handling ambiguous cases can be effectively positioned along their ROC curve. (Bottom) When data is further broken into easy and hard cases, easy examples see moderately increased agreement and hard examples see more disagreement, resembling the human agreement histogram of Figure 4.

Based on our experiences, we would recommend taking time to discuss questionable examples and reasons for rating them one way or another. Such conversations are rather illuminating and very effective at getting everyone to agree on a common standard of classification.

### Limitations and Future Work

There are two significant limitations to the questions which can be asked using this method. The first and strictest: an array tomography volume is a decidedly terminal snapshot of a piece of tissue. This precludes experiments which would watch a particular cell or dendrite change over time, or in response to learning [34], except in animal models which are stereotyped enough for different animals to have equivalent nervous systems, namely C. Elegans [35] and Drosophila [36]. Synapse populations are assumed to be fairly invariant between individual mice (and presumably humans), however, which allows us to study changes to synaptic classes as a whole in response to plasticity or disease.

The second limitation is more easily rectified. Our analysis partially depends on limiting the scope of the problem to that required to identify synapses at locations already suspected to contain a synapse. For common synapse classes this is easy. They all express Synapsin I, so wherever we find our Synapsin I marker, there may be a synapse. As mentioned, we have already begun to abut the usefulness of Synapsin I, which may not be expressed in dopaminergic synapses [25]. Using a pan-Synapsin antibody would be a straightforward solution to catching all dopaminergic synapses, but it is fully possible that other, more exotic synapse types may not express Synapsin at all, instead relying on some currently unknown mechanism to perform the same function.

Establishing a robust system for synapse classification in array tomographic volumes opens up a number of avenues for addressing biological questions. It allows us to conduct single-synapse analyses in large regions of tissue, which lets us study rare or spatially-segregated populations. It helps us discover new synaptic populations and novel variations on known synapse types, and gives us an unprecedented level of control over the proteomic complexity we can bring to bear.

## Materials and Methods

### Acquisition of Array Tomographic Volume

All procedures related to the care and treatment of animals were approved by the Administrative Panel on Laboratory Animal Care at Stanford University. All volumes were acquired from mouse cortex, line C57BL/6J, using the methodology given in [17].

One adult mouse was used for this study. The animal was anesthetized by halothane inhalation and its brain quickly removed and placed in 4% formaldehyde and 2.5% sucrose in phosphate-buffered saline (PBS) at room temperature. Its cerebral hemisphere was sliced coronally into three pieces and fixed and embedded using rapid microwave irradiation (PELCO 3451 laboratory microwave system with ColdSpot; Ted Pella, Redding CA) as described in [37]. The tissue was dehydrated up to 70% ethanol.

Ribbons of serial ultrathin (70 nm) sections were cut with an ultramicrotome (EM UC6, Leica Microsystems, Wetzlar, Germany) as described in [37]. The ribbons were mounted on subbed coverslips (coated with 0.5% gelatin and 0.05% chromium potassium sulfate) and placed on a hot plate (60 C) for 30 min. For SEM imaging, the subbed coverslips were also carbon coated using a Denton Bench Top Turbo Carbon Evaporator (Denton Vacuum, Moorestown, NJ). Subbed and carbon coated coverslips were also prepared for mounting ribbons of sections to be used for multiple immunostaining rounds (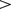
6).

Staining was performed as described in [37]. The coverslips with sections were mounted using SlowFade Gold antifade with DAPI (Invitrogen, Carlsbad CA). To elute the applied antibodies, the mounting medium was washed away with dH2O and a solution of 0.2 M NaOH and 0.02% SDS in distilled water was applied for 20 min. After an extensive wash with Tris buffer and distilled water, the coverslips were dried and placed on a hot plate (60C) for 30 min.

The primary antibodies and their dilutions are listed in [17], Table 1. Only well characterized commercial antibodies were used and they were evaluated specifically for AT as described in the Supplemental Experimental Procedures of [17]. For immunofluorescence, Alexa Fluor 488, 594, and 647 secondary antibodies of the appropriate species, highly preadsorbed (Invitrogen, Carlsbad CA) were used at a dilution 1∶150. The sequence of antibody application in the multiround staining is presented in [17], Table S1.

Sections were imaged on a Zeiss Axio Imager.Z1 Upright Fluorescence Microscope with motorized stage and Axiocam HR Digital Camera as described in [37]. Briefly, a tiled image of the entire ribbon of sections on a coverslip was obtained using a 10 objective and the MosaiX feature of the software. The region of interest was then identified on each section with custom-made software and imaged at a higher magnification with a Zeiss 63/1.4 NA Plan Apochromat objective, using the image-based automatic focus capability of the software. The resulting stack of images was exported to ImageJ, aligned using the MultiStackReg plugin and imported back into the Axiovision software to generate a volume rendering. When a ribbon was stained and imaged multiple times, the MultiStackReg plugin was used to register the stacks generated from each successive imaging session with the first session stacks based on the DAPI channel, then a second within-stack alignment was applied to all the stacks. Since DAPI was stained in all imaging sessions it made an ideal candidate for alignment, and the alignment transformation of each imaging session's DAPI channel was propagated to the other members of that session to bring the entire channel set into the same coordinate space.

To reconstruct the larger volume of tissue used in this study, we first used Zeiss Axiovision software to stitch together individual high-magnification image tiles and produce a single mosaic image of each antibody stain for each serial section in the ribbon, creating a z stack of mosaic images for each fluorescence channel instead of a single field of view stack. To coarsely align the image stacks, we used the MultiStackReg plugin with the DAPI channel, as described above and in [37].

To analyze synapse-level structures an additional alignment step was needed to remove a minor non-linear physical warping introduced into the ribbons by the sectioning process. We used a second ImageJ plugin, autobUnwarpJ (available at http://www.stanford.edu/~nweiler), which adapts an algorithm for elastic image registration using vector-spline regularization [38]. As before, we aligned only a single channel, Synapsin, and propagated the generated transformation to the other channels. Synapsin proved ideal for this purpose because it is a dense, high-frequency channel whose labeled objects are still considerably thicker than a single section, creating good fiducial markers for the alignment process.

Finally, data used for Table 3 and Figure S2 were processed after imaging using a method of deconvolution recently published by our lab [39]. This does not seem to affect MLA performance, but the smaller, more discrete puncta do cause an increase in the number of synapsin local maxima, and therefore generates more extracted synapsin loci. Future work using deconvolved volumes may benefit from incorporating an additional filtering step in the extraction process to either smooth the data before finding local maxima or segment puncta more directly.

### Normalization and Background Subtraction of Volumetric Data

Before analyzing imaged volumes, we subtracted the background from each fluorescent channel using a 10×10 pixel (1 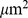) rolling ball filter to remove systematic non-punctate background fluorescence, then normalized each slice of the stack without saturating any pixels, such that the brightness histogram of each section was stretched as much as possible without loss of information. No other image processing, including removal of fluorescence due to foreign material, nonspecific staining, etc, was performed before analysis.

### Extraction of Synaptic Loci

To extract a list of putative synapse locations from raw volume data, we first identified individual synapsin puncta by convolving the synapsin channel with a 3×3×3 local maxima filter; retaining all voxels with a brightness 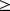 those of its 26-voxel neighborhood. Then, we passed the synapsin maxima through a connected component filter to reduce peak voxel clumps (caused by discretization of the fluorescence data) to centroids, and discarded those below a deliberately low threshold (10% of the total brightness range) as being too dim to represent a real synapse. What remained was a list of putative synapse locations, or “synaptic loci,” so named for their central role in later classification steps.

### Estimating Human Rater Agreement

In order to gauge the reliability of any single human expert's rating, we performed a qualitative test of the consistency of human classification. We presented a set of one hundred randomly-selected synaptograms to a group of six human raters who were familiar with the task of interpreting synaptograms, and instructed them to classify the set based on whether or not the synaptogram was centered on a glutamatergic synapse. Once collated, we considered the true classification of a given synaptogram to be that of a simple majority vote of the first five raters (to prevent ties). When we compared each rater's performance relative to the average, we found an average accuracy rate of 77.7%, with a standard deviation of 10.1% (Figure 4). The largest source of variance arose from the self-reported stringency of the raters, in how much ambiguity they found acceptable when classifying a locus as positive.

### Estimating MLA Agreement

To test the influence that training stringency and classification difficulty have on MLA performance, we repeated the above test with three classifiers trained by rater 1. In addition to the classifier trained with the default strategy, classifiers that would attempt to guess “yes” or “no” in ambiguous cases were trained and their mutual performance compared, using their average agreement to establish a gold standard as was the case for the humans. We also subdivided the data set further into “easy” versus “hard” cases through the use of a fourth MLA, and compared those conditions as well.

### PCA Image Treatment

The color of each point in the PCA figure was determined by taking the extreme outliers of the three clusters, determining their feature composition via multivariate regression, taking the dot product of the feature weight vectors with the feature vector of each locus, and assigning that to red, green or blue for the VGluT1, VGluT2, and GABA clusters respectively. Colors were manually normalized to be of approximately equal intensity, and synaptic loci not strongly represented in any of the three colors were removed to better visualize cluster relationship.

### MLA Training Strategy

The training of our machine learning algorithm differs from standard supervised learning, in which training examples are chosen at random, by instead selecting examples which together compose a varied training set. We also added a preprocessing step to facilitate the training of very rare classes, on our case VAchT and VGluT3. Thus the training set generation occurs in two phases. The first phase is to “prime” the training set data for rare classes by choosing one of each class's requisite presynaptic channels and randomly sampling a subset from the loci for which the channel's local brightness is more than two standard deviations above the mean. A number of class subsets generated in this manner are collated, each class contributing to the negative examples of the rest. The second phase is an “active” training process in which a human rater and the MLA being trained work in tandem to speed training, a technique known as active learning [40], [41]. At each step, the half-trained classifier selects a few examples, half of which it thinks are positive and half negative, to present to the rater for verification and feedback.

In pseudocode, the training proceeds according to the following algorithm:

**while** Human wishes to train **do**

 Load training synaptogram population, 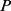


 Human selects a synaptic category

 Train RFE using partially classified training set 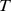, display predicted error rate

**while** Human wishes to add training examples **do**

  Randomly choose 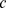, where 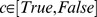


  Randomly choose a synaptogram 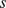 from subpopulation 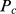, the elements of 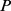 classified as 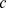


  Display 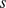 and 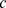 to human for verification

  Add/Update 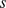 in 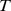 to reflect human input

**end while**

**end while**

### Normalization of Pairwise Channel Data

To produce the pairwise channel copresence map, for each marker pair 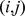 we calculated the probability of co-occurrence 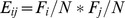, where 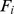 is the number of loci found to be positive for 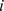, and 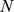 is the number of total loci in the population. Multiplying by 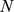 gives us the expected population, 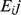. We compared this number with the observed population 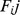 using difference over sum normalization to find the normalized pairwise relationship 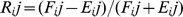. These relationships made pairwise comparisons easy to interpret, with one minor counter-intuitive exception: markers which comprised a substantial proportion of the synaptic loci population (VGluT1 and PSD95) had reduced values, even with themselves, owing to their high 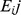. To bring those into the same reference frame as the rest, we normalized again using the reciprocal of the sum of the relationship identity reciprocals, that is, 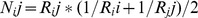. Finally, since the previous step disrupted negative relationship scaling such that the most negative pairs (VGluT1 vs GABAergic markers) reached nearly −3.0, we multiplied the positive ratings by 3 to match once more.

### Perpendicularization of Cortical Data

To simplify the calculation of the cortical depth-dependent metrics used in Figure 5, such that any given Y-value represented tissue at the same cortical depth, we needed to correct a minor slant in the raw volume. We measured the degree of tissue slope using the pial surface and the white matter/striatum boundary, and imposed an affine transformation on the loci, linearly interpolating them to be level. The underlying data and the features used to classify the loci were not changed as a result of this process.

### Software Packages Used

Image normalization, locus discovery and feature extraction were implemented and performed using Fiji (http://fiji.sc/). Training set generation was implemented as a browser-based application, coded in Python, to permit our experts to work at their leisure. We used R for interactive classification for its ease of Python integration, but the final random forest classifiers, trained on the complete training set alone, used MATLAB (the TreeBagger class). Imaris was used to render the data for visualization of Figure 1.

All implemented code used in this analysis is available at http://code.google.com/p/smithlabsoftware/ under a GPL v3 license.

## Supporting Information

Figure S1
**Erythrocyte diameter as indicator of tissue shrinkage.** (A) A single 200 nm section from the same block of prepared tissue as the full cortical depth analysis, displaying autofluorescent red blood cells with a maximum diameter of 4.6 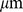 (arrows). (B) Fresh erythrocytes have a diameter of 6 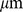 (arrows), indicating a linear shrinkage of 23% and a volumetric shrinkage of 54% as a side effect of tissue dehydration. Scale bar 2 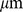.(TIF)

Figure S2
**Layer 3/4 VGluT2+ synapse distribution.** Processed in the same manner as the full depth analysis of Figure 5, VgluT2+ synapses display a similar peak density at the boundary between layer 3 and 4. The two populations, shown here separately for clarity, were taken from adjacent fields of view with an approximately 10% overlap. Each bin represents 10 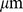 of cortical depth.(TIF)
