# Supplementary figures and images for: Automated Analysis of a Diverse Synapse Population
Source: PLoS Comput Biol. 2013 Mar 28;9(3):e1002976. doi: 10.1371/journal.pcbi.1002976 (PMC3610606; doi:10.1371/journal.pcbi.1002976)

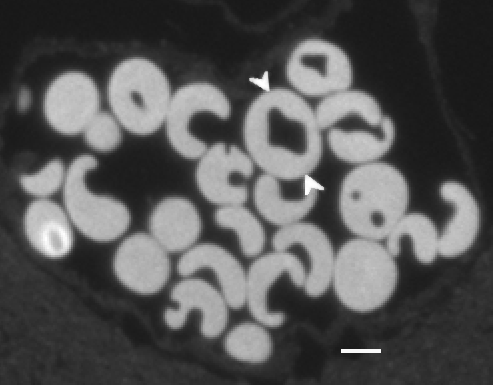

Supplement: Figure S1 — Erythrocyte diameter as indicator of tissue shrinkage. (A) A single 200 nm section from the same block of prepared tissue as the full cortical depth analysis, displaying autofluorescent red blood cells with a maximum diameter of 4.6 (arrows). (B) Fresh erythrocytes have a diameter of 6 (arrows), indicating a linear shrinkage of 23% and a volumetric shrinkage of 54% as a side effect of tissue dehydration. Scale bar 2 . (TIF) [file pcbi.1002976.s001.tif]

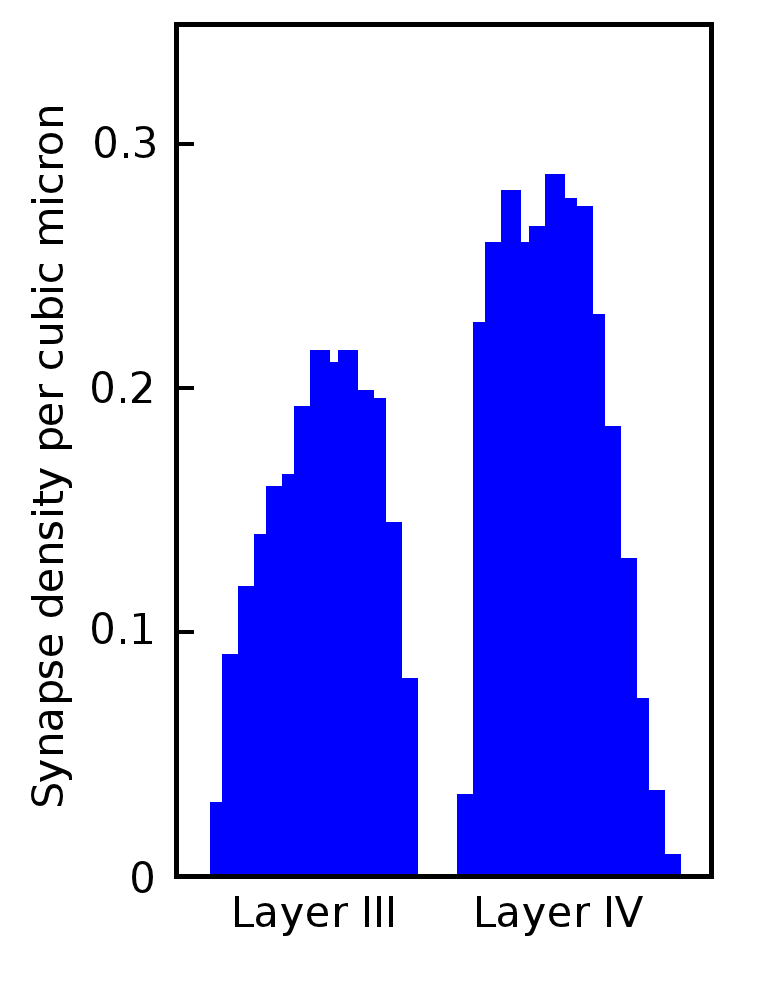

Supplement: Figure S2 — Layer 3/4 VGluT2+ synapse distribution. Processed in the same manner as the full depth analysis of Figure 5, VgluT2+ synapses display a similar peak density at the boundary between layer 3 and 4. The two populations, shown here separately for clarity, were taken from adjacent fields of view with an approximately 10% overlap. Each bin represents 10 of cortical depth. (TIF) [file pcbi.1002976.s002.tif]
